# High efficiency cabin air filter in vehicles reduces drivers' roadway particulate matter exposures and associated lipid peroxidation

**DOI:** 10.1371/journal.pone.0188498

**Published:** 2017-11-27

**Authors:** Nu Yu, Shi Shu, Yan Lin, Jianwen She, Ho Sai Simon Ip, Xinghua Qiu, Yifang Zhu

**Affiliations:** 1 Department of Environmental Health Sciences, Jonathan and Karin Fielding School of Public Health, University of California, Los Angeles, Los Angeles, California, United States; 2 California Department of Public Health, 850 Marina Bay Parkway, Richmond, California, United States; 3 State Key Joint Laboratory for Environmental Simulation and Pollution Control, College of Environmental Sciences and Engineering and Center for Environment and Health, Peking University, Beijing, People’s Republic of China; University of Louisville School of Medicine, UNITED STATES

## Abstract

Commuters who spend long hours on roads are exposed to high levels of traffic related air pollutants (TRAPs). Despite some well-known multiple adverse effects of TRAPs on human health, limited studies have focused on mitigation strategies to reduce these effects. In this study, we measured fine particulate matter (PM_2.5_) and ultrafine particle (UFP) concentrations inside and outside 17 taxis simultaneously while they were driven on roadways. The drivers’ urinary monohydroxylated polycyclic aromatic hydrocarbons (OH-PAHs) and malondialdehyde (MDA) concentrations just before and right after the driving tests were also determined. Data were collected under three driving conditions (i.e. no mitigation (NM), window closed (WC), and window closed plus using high efficiency cabin air filters (WC+HECA)) for each taxi and driver. The results show that, compared to NM, the WC+HECA reduced in-cabin PM_2.5_ and UFP concentrations, by 37% and 47% respectively (p < 0.05), whereas the reductions on PAH exposures were insignificant. Although nonsignificant, a reduction of 17% was also observed in the drivers’ urinary MDA under WC+HECA. The MDA concentrations were found to be significantly associated with the in-cabin PM_2.5_ and UFP concentrations, suggesting the reduction of the drivers’ lipid peroxidation can be at least partially attributed to the PM_2.5_ and UFP reduction by WC+HECA. Overall, these results suggest HECA filters have potential to reduce particle levels inside taxis and protect drivers’ health.

## Introduction

Air pollution is one of the leading risk factors of human premature death globally [1, 2]. As the most substantial combustion source in urban areas with dense population, vehicular emissions significantly increase carbon monoxide (CO), nitrogen oxides (NOx), fine particulate matter (PM_2.5_), ultrafine particles (UFPs), and polycyclic aromatic hydrocarbon (PAH) concentrations on/near roadways and inside vehicles [3–6]. Over 600 million people worldwide are exposed to these hazardous traffic related air pollutants (TRAPs) [7, 8], which have been associated with various adverse health effects [9–11].

Among the TRAPs, PM_2.5,_ UFP and PAHs have been found to be associated with cardiovascular diseases, which are the primary contributors to the air pollution related premature deaths [12]. Because of the frequently observed association between PM_2.5_ and the oxidative damage, the oxidative stress has been suggested to play a role linking the exposures with the cardiovascular diseases, although the mechanism has not been fully understood. Specifically, previous studies have shown that PM_2.5_ and UFP from traffic emissions have high oxidative potential that induces elevated systematic oxidative stress in rats and human [13–15]. Malondialdehyde (MDA) is a lipid peroxidation end-product and is usually produced under body systematic oxidative stress. Some *in vitro* and *in vivo* studies also suggest that PAHs and their metabolites can join the redox-active cycle and produce reactive oxygen species (ROS), which can attack larger biological molecules including polyunsaturated lipids and generate MDA [16, 17]. In addition, MDA has the potential to react with nucleic acid bases to form DNA adducts, create DNA interstrand, and even DNA protein cross-links to induce more health risks [18, 19]. Thus, MDA is a widely used biomarker for evaluation of oxidative stress associated with exposures to combustion-related air pollutants [18, 20].

Vehicular emissions are known as the primary sources of ambient PM in the Los Angeles air basin [21]. About 33–45% of the total UFP exposure for Los Angeles residents occur due to time spent traveling in vehicles [22]. Some mitigation strategies have been explored in previous studies to reduce PM exposures for commuter, which include closing vehicle windows and using the high efficiency cabin air (HECA) filters. A passenger vehicle study found that keeping vehicle windows closed and using outside air ventilation mode reduced the on-road PM by up to 40% [23]. Furthermore, when the vehicle was equipped with a HECA filter, it reduced the on-road PM_2.5_ and UFP by 70% and 92%, respectively [24]. However, it remains unknown to what extent these mitigation strategies benefit the commuters’ health.

About 4,000 taxi drivers are working in the Greater Los Angeles area. They work 72 hours per week on average, and potentially have high TRAP exposures because of the long hours spent on roadways [25]. In this study, we measured real time PM_2.5_ and UFP concentrations simultaneously inside and outside 17 Los Angeles taxis while they were driven on roads, under different mitigation conditions. The drivers’ urinary monohydroxylated-PAHs (OH-PAHs) and MDA concentrations were also measured just before and right after each 6-hour monitored driving test. The aim of this study is to evaluate whether using HECA filters can effectively reduce the drivers’ PM and PAH exposures, as well as the lipid peroxidation levels in their body.

## Methods

### Subjects

The detailed description on the taxi driver recruitment can be found elsewhere [26]. Briefly, in March 2013, 2449 recruitment forms were hand distributed to taxi drivers passing through the Los Angeles International Airport (LAX) taxi holding lot. A total of 316 completed forms were obtained. Taxi drivers who smoked or drove Ford Crown Victoria taxis were excluded from this study, because cigarette smoking interferes with the PAH measurements, and the Ford Crown Victoria taxis don’t have a cabin air filter holder. Finally, 17 subjects were random picked out of the 90 qualified taxi drivers. Their demographic characteristics, body mass index (BMI), years as taxi drivers, and taxi vehicle make/models are summarized in Table 1. The study design and experimental protocol have been approved by the UCLA Institutional Review Board (IRB). Informed consents were obtained from all participants.

**Table 1:** Characteristics of the studied taxi drivers*.

	Mean ± SD	Minimum	Maximum
**Age (year)**	47 ± 13	28	67
**Year as Taxi Driver**	10 ± 6	2	20
**BMI (kg / m**^**2**^**)**	26.8 ± 4.6	19.5	38.7
**Ethnicity, n (%)**	**Taxi Model, n (%)**		
*Black*	2 (11.8%)	*Chevy Uplander*	1 (5.9%)
*Hispanic/Latino*	0 (0%)	*Dodge Caravan*	3 (17.6%)
*White*	6 (35.3%)	*Toyota Prius*	10 (58.8%)
*Asian*	5 (29.4%)	*Toyota Camry*	3 (17.6%)
*Other*	(23.5%)		

* Of seventeen total, sixteen were males (94.1%) and one female.

### Monitoring of driving test

Each driver was monitored for six hours a day and for three consecutive days while driving in the Greater Los Angeles area. The starting time and driving routes of each test day were kept consistent for each driver. During the tests the drivers were requested to mimic their actual work for driving, parking/waiting, and taking breaks during the monitored six hours.

On the first test day, the taxi drivers were allowed to control the window positions and use their originally equipped manufacturer (OEM) cabin filter to simulate their regular working conditions without any mitigation (No mitigation; NM). On the second test day, the taxi drivers were requested to keep their vehicle windows closed, but their OEM cabin filter remained unchanged (Window closed; WC). The third test day was designed to have the most strict mitigation strategy in place by keeping the taxi windows closed and using a HECA filter simultaneously (WC+HECA). The HECA filters were provided by an industrial partner and technical details of the filters are described elsewhere [24]. Throughout the period of measurements, the taxi ventilation system was set to outdoor air mode to avoid CO_2_ build-up. The fan was set to medium level when the windows were closed. The total mileage driven by the tested taxi drivers was approximately 11,000 kilometers and the total amount of time of field measurements was approximately 300 hours.

### PM monitoring and urine collection

During the 6-hr monitored driving tests, two sets of portable condensation particle counter (CPC) (Model 3007, TSI Inc., St. Paul, MN) and DustTrak (Model 8520, TSI Inc., St. Paul, MN) were deployed to measure UFP and PM_2.5_ concentrations concurrently inside and outside of each taxi, respectively. The CPC measures the number concentration for particles sizes from 0.01 to 1.0 μm. The DustTrak was connected with a 2.5 μm impactor to measure the PM_2.5_ mass concentration. For in-cabin measurements, the inlets of the two pieces of instrument were connected with the same length of tubing extending to the driver’s breathing zone. For on-road measurements, similar tubing of the same length was mounted on the taxi window to compensate any diffusion loss in the sampling lines. Both CPC and DustTrak are real-time direct reading instrument with data logging function. UFP and PM_2.5_ concentrations were logged with 1-sec interval during the experiments. Daily 6-hr averages were used for subsequent analysis. Urine samples were collected in 90 ml sterile screw cap urine containers just before and right after the 6-hr monitored driving test (pre- and post-test) on each test day for each taxi driver. All collected urine samples were stored in a -20°C freezer until analysis.

### Analysis of urinary OH-PAHs and MDA

The OH-PAH analysis method was based on enzymatic deconjugation, liquid-liquid extraction, trimethylsilylation of the OH-PAHs, followed by gas chromatography/isotope dilution high-resolution mass spectrometry [27]. Nine OH-PAHs quantified were: 1- and 2-hydroxynaphthalene (1- and 2-OH-NAP), 2-, 3- and 9-hydroxyfluorine (2-, 3-, and 9-OH-FLU), 1-, 2-, and 3-hydroxyphenanthrene (1-, 2-, and 3-OH-PHE), and 1-hydroxypyrene (1-OH-PYR). Explained in a previous study [28], briefly, the urinary MDA was analyzed by a high performance liquid chromatography (HPLC) system with a fluorescent detector. Each urine sample was added with a mixture of phosphoric acid and thiobarbituric acid (TBA, 42 mM), and incubated for 1 hour at 80°C. Then the solution with MDA-TBA derivatives was injected into the HPLC system with a fluorescence detector at 532 nm. Urinary creatinine was analyzed with Jaffe method which generates stable creatinine results for urine samples kept at a temperature of -20°C and thawed only once [29, 30]. The urinary OH-PAH and MDA concentrations were adjusted with creatinine.

### Quality assurance and quality control

For both CPC and DustTrak, flow rate measurement and zero check with HEPA filters was performed before and after each monitored tests. The DustTrak monitor was calibrated against simultaneous gravimetric measurements of PM_2.5_. A factor of 2.4 was achieved and used for DustTrak data correction, which was consistent with data reported in previous studies [31, 32]. For OH-PAH analysis, quality control (QC) samples were analyzed along with the taxi driver samples to assure the precision and accuracy of the data. QC data were reviewed based on Westgard rule [33, 34]. Proficiency Test (PT) or inter-laboratory QC samples of unknown concentrations were performed twice a year for demonstration of OH-PAH analytical method performance.

### Estimation of PAH exposure due to traffic

Urinary OH-PAHs are biomarkers of integrated body PAH intake through different exposure routes. The half-lives of the urinary OH-PAHs are usually several hours in human body, and it takes days to completely excrete OH-PAHs after the PAH exposures. Although we tried to minimize the PAH intake from other sources by prohibiting eating barbequed or fried food during the test days, taxi drivers’ urinary OH-PAH levels could still be influenced by the PAH exposures other than the traffic emissions during the monitored driving tests, such as previous TRAP exposures or dietary intake. Therefore, neither the pre- nor post-test urinary OH-PAHs can directly indicate the traffic-related PAH exposure levels during the monitored taxi driver tests. Instead, we used a well-established one-compartment pharmacokinetic model with continuous infusion to estimate the traffic-related PAH exposure during the monitored tests [35]. In this model, we assumed constant and continuous exposure to PAHs during the tested six hours. We also assumed PAHs underwent a first-order elimination in human body, which is widely supported by previous studies [36]. The average elimination rates of different PAH species were obtained from a previous study [37]. The details of the calculation method are showed in Section A of S1 File. The calculated OH-PAH increments, denoted as OH-PAH_trap_, were used as the surrogates of the traffic-related PAH exposure during the driving tests.

### Data and statistical analysis

The urinary OH-PAH concentrations below detection limits (BDL) were calculated as half of the detection limit values. Calculated urinary OH-PAH increments due to the TRAP exposure during the monitored tests (OH-PAH_trap_) were used as the surrogates of traffic-related PAHs exposure during the monitored six hours. In order to normalize the variable distributions, all sampling results were log transformed. Shapiro-Wilk test was conducted to ensure the transformed data were normally distributed before running the subsequent statistical tests and regression models.

Paired t-test was used to detect differences for in-cabin and on-road UFP and PM_2.5_ levels as well as OH-PAH_trap_ between interventions and the NM condition. Pearson’s correlations and mixed effect linear regression models with random intercepts for each driver were used to test the associations between variables. The model can be expressed by the equation below:

$$y_{ij}=\alpha +\mu_{i}+\beta x_{ij}+\varepsilon_{ij}$$

Where *i* is the index of each driver, and *j* is the index of mitigation strategies. α and β are the fixed intercept and slope respectively, and μ_*i*_ is the random intercept of each driver under each mitigation condition. ε_*ij*_ is the residual. PM_2.5,_ UFP, and OH-PAH_trap_ were log transformed before fitting the model. The level of significance was taken as *p < 0*.*05*. SAS 9.4 software (SAS Institute, Cary, NC) was used for statistical analysis.

## Results

### On-road and in-cabin PM and PAH exposures

The PM_2.5_ and UFPs concentrations measured inside and outside the 17 taxis during the driving tests were summarized in Table 2. Because the start time, driving routes, and break times were well controlled, and the driving tests were conducted in three consecutive days to test the three mitigation conditions for each driver, the on-road PM_2.5_ and UFPs didn’t show significant differences based on the paired t-test among different test conditions.

**Table 2:** Summary of taxi drivers’ exposures under different test conditions (geometric mean, interquartile range, and percentage of change from NM).

Mitigation	NM^a^	WC^b^	WC+HECA^c^
**On-road PM**_**2.5**_ **(**μ**g/m**^**3**^**)**	31 (20, 53)	28 (20, 40) (-10%)^d^	29 (24, 33) (-6%)
**In-cabin PM**_**2.5**_ **(**μ**g/m**^**3**^**)**	19 (15, 22)	20 (16, 20) (5%)	12 (10, 16) * (-37%)
**On-road UFP** **(x10**^**4**^ **cm**^**-3**^**)**	2.71 (2.44, 3.02)	2.72 (2.29, 3.46) (0%)	2.70 (2.34, 3.55) (-0%)
**In-cabin UFP** **(x10**^**4**^ **cm**^**-3**^**)**	1.40 (1.13, 1.97)	1.33 (1.07, 1.77) (-5%)	0.74 (0.59, 1.02) * (-47%)
**∑OH-NAP**_**trap**_ **(**μ**g/g cr)**	4.69 (2.30, 8.96)	3.47 (1.99, 5.38) (-26%)	4.40 (2.50, 6.47) (-6%)
**∑OH-FLU**_**trap**_ **(**μ**g/g cr)**	0.51 (0.29, 0.71)	0.40 (0.30, 0.74) (-22%)	0.48 (0.33, 0.60) (-6%)
**∑OH-PHE**_**trap**_ **(**μ**g/g cr)**	0.15 (0.08, 0.24)	0.12 (0.07, 0.20) (-20%)	0.18 (0.14, 0.20) (20%)
**1-OH-PYR**_**trap**_ **(**μ**g/g cr)**	0.05 (0.02, 0.10)	0.05 (0.02, 0.10) (0%)	0.07 (0.05, 0.10) (40%)
**Pre-test MDA** **(μg/g cr)**	20.80 (20.53, 54.18)	10.74 (9.23, 40.95) (-48%)	27.14 (13.49, 56.39) (30%)
**Post-test MDA** **(μg/g cr)**	31.91 (22.78, 47.25)	31.73 (22.87, 43.90) (-1%)	23.68 (9.99, 55.37) (-17%)

a. No mitigation

b. Window closed

c. High efficiency cabin air filter

d. Numbers in parenthesis indicate percentage of change from NM.

* indicate significance of paired t-test (*p* < *0*.*05*) compared with NM.

Under the NM condition, the vehicle ventilation and window position were controlled by the drivers. The sampling results reflect their exposures to UFP, PM_2.5_, and PAHs under normal working conditions. The geometric mean PM_2.5_ and UFPs levels inside the tested 17 taxis were 19 **μ**g/m^3^ and 1.40 x 10^4^ particles/cm^3^, respectively. The model calculated increments due to traffic exposure for ∑OH-NAP, ∑OH-FLU, ∑OH-PHE and 1-OH-PYR were 4.69, 0.51, 0.15, and 0.05 **μ**g/g creatinine (cr), respectively (Table 2).

Compared with the NM condition, WC condition changed the in-cabin geometric mean PM_2.5_ and UFP by -5% and 5%, respectively, but the differences were not significant (*p* > *0*.*05*). WC did not reduce the taxi driver urinary OH-PAH_trap_ of any individual PAH species either (Table 2). However, under WC+HECA, compared with NM, the geometric mean PM_2.5_ and UFP levels were reduced by 37% and 47%, respectively. The geometric mean ∑OH-NAP_trap_ and ∑OH-FLU_trap_ levels were reduced by 6%, whereas the geometric mean ∑OH-PHE_trap_ and 1-OH-PYR_trap_ levels increased 20% and 40%, respectively. The reduction of PM_2.5_ and UFP concentrations were significant *(p < 0*.*05)* (Table 2 and Table A in S1 File). These results show that, as a mitigation strategy, WC+HECA is effective in reducing PM levels inside these tested 17 taxi vehicles, but not effective in reducing urinary PAH metabolize levels of taxi drivers.

Table 2 also shows that, the geometric mean post-test MDA levels were similar under NM and WC (1% difference), but was reduced by 17% under the WC+HECA condition. Although the reduction was not significant (*p* > *0*.*05*), the trend among the three mitigation conditions was similar with the trends of in-cabin PM_2.5_ and UFP concentrations, suggesting the changes in MDA might be linked with PM_2.5_ and UFP reduction. This hypothesis is tested in the following section.

### Associations among PM, OH-PAHs, and MDA

The in-cabin geometric mean PM_2.5_ concentrations were not significantly correlated with UFP or any calculated OH-PAH_trap_ levels. However, the geometric mean UFP concentrations were correlated significantly with 1-OH-PYR_trap_ levels (*p* < 0.05). For each OH-PAH species, significant correlations between pre- and post-test levels were detected (*p* < 0.05, Table B in S1 File), suggesting the impacts of pre-test OH-PAH levels on the post-test OH-PAHs. Therefore, the post-test OH-PAH levels were not good indicators of the taxi drivers’ in-cabin PAH exposures during the driving tests. Instead, the calculated OH-PAH_trap_ levels were used as the surrogate of traffic-related PAH exposures during the 6-hr driving test in this study.

Unlike the urinary OH-PAHs, the pre- and post-test MDA concentrations didn’t show significant correlation with each other. However, the post-test MDA levels showed a similar trend with PM and OH-PAH_trap,_ in that the lowest geometric mean was observed under WC+HECA (Table 2). Thus, the TRAP exposures might have some impacts on the post-test MDA levels.

**Fig 1:**
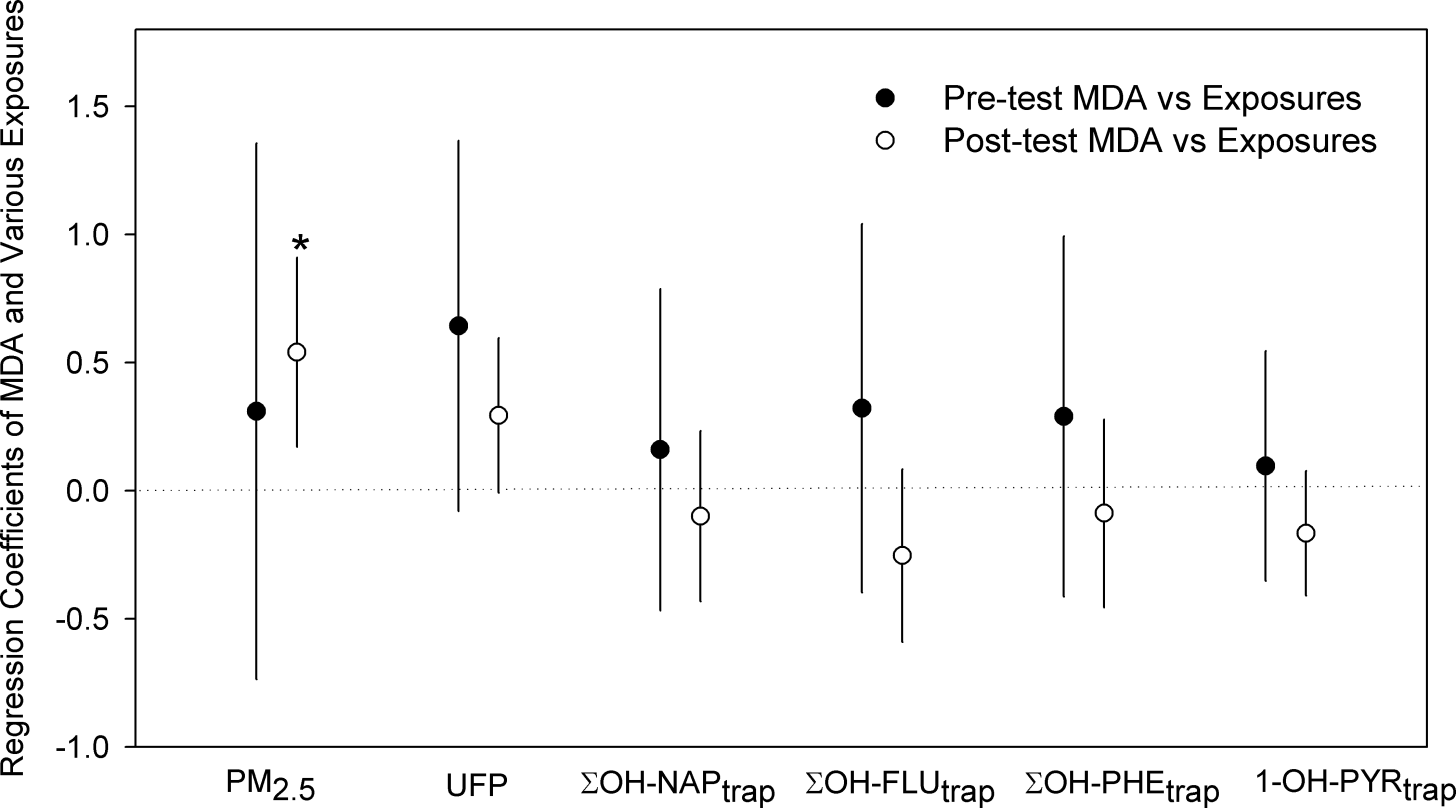
Regression coefficients of pre- and post-test urinary MDA and various exposure indicators including PM_2.5_ and UFP levels inside taxi as well as PAH metabolites in urine. * indicates significant correlations *(p < 0*.*05)*, error bars indicate 95% confidence intervals.

To examine the association between PM and PAH exposures and MDA, a mixed effect linear model was developed. Other factors that might affect the MDA levels, such as temperature, relative humidity, and weekday/weekend, were also included to the initial models, but the results showed that they were not significant, and didn’t affect the associations between exposures and the MDA. Thus the final models only include exposures and individual effects. Linear regression coefficients and their confidence intervals were summarized in Fig 1 to show the significance of the correlations at *α = 0*.*05* level. The taxi driver post-test urinary MDA levels were observed to have significant positive correlations with their in-cabin PM_2.5_ exposure during the 6-hr driving test, and have marginally significant positive correlations with their in-cabin UFP levels *(p = 0*.*07)* (Fig 1).

## Discussion

### Taxi driver PM and PAH exposures

The PM_2.5_ and UFP levels measured inside taxis under NM were on the similar levels with those measured inside private vehicles [3, 23]. However, since taxi drivers, on average, drive for 12 hours on each working day, which composes of 50% of their daily activities, their total exposures to roadway PM_2.5_ and UFPs are about six times higher than other Southern California regular commuters, given that an average commuter in Southern California spends 7.2% of his/her day driving in traffic [38].

**Fig 2:**
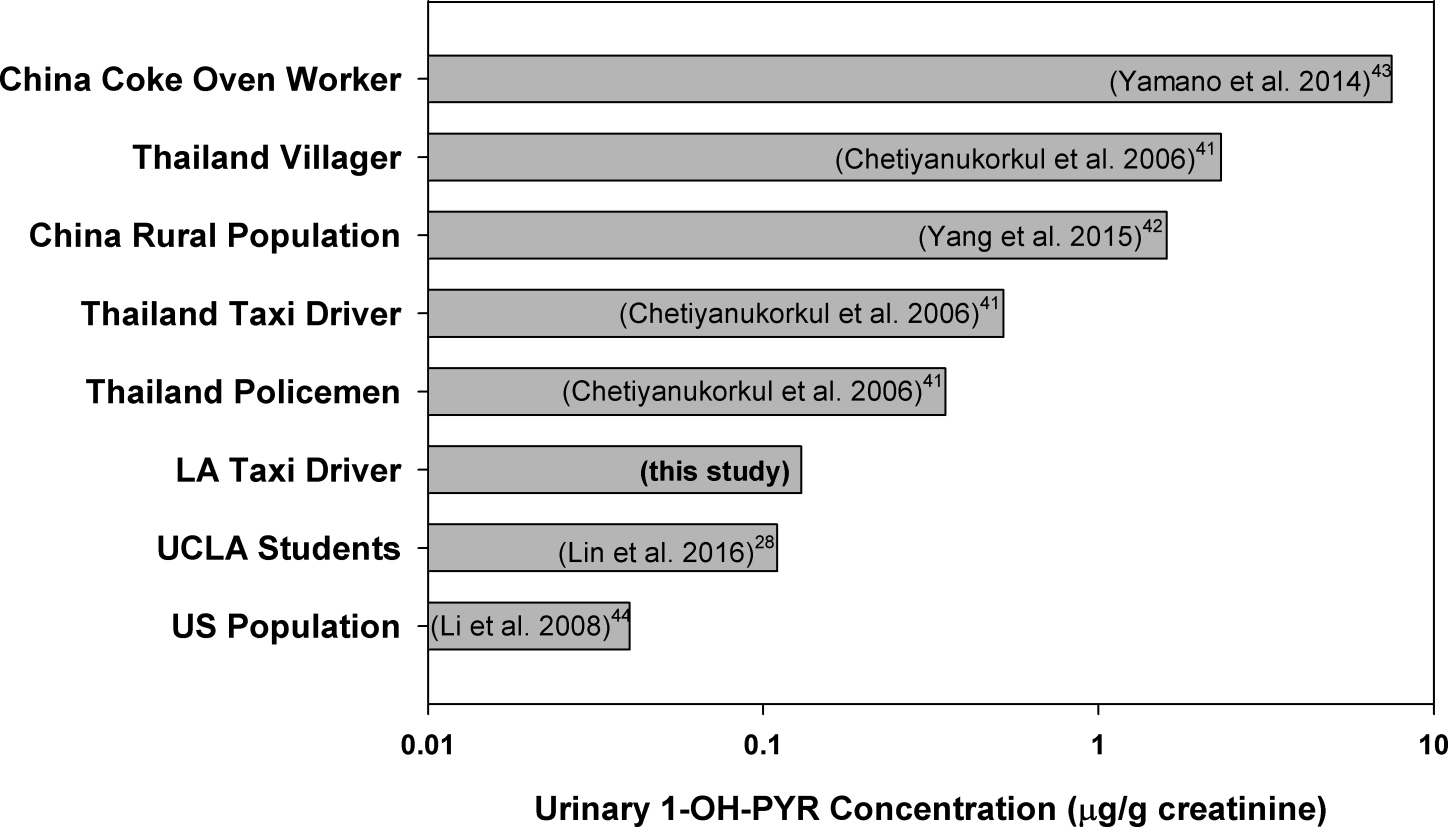
Comparison of Los Angeles taxi driver urinary 1-PYR concentrations with literature data from U.S. population (NHANES), UCLA students, and some other occupational groups.

Since urinary 1-OH-PYR is a widely used biomarker for human total PAH exposure assessments [39, 40], urinary 1-OH-PYR concentration was used to compare the taxi drivers with other population. The results are illustrated in Fig 2. The median concentration of 1-OH-PYR of this taxi driver group were about 125% higher than the U.S. general population median from National Health and Nutrition Examination Survey (NHANES), 50% higher than a group of University of California Los Angeles (UCLA) students [28], but lower than taxi drivers or other occupational groups in Thailand and China [41–44].

### Exposure mitigation

In this study, only 2-ring (NAP), 3-ring (FLU and PHE), and 4-ring (PYR) PAHs were measured by their urinary monohydroxylated metabolites. In all collected urine samples, on average, the two OH-NAPs (2.28 and 5.20 **μ**g/g cr) were most abundant, followed by the three OH-FLUs (0.13–0.42 **μ**g/g cr), the three OH-PHEs (0.06–0.15 **μ**g/g cr), and the 1-OH-PYR (0.13 **μ**g/g cr). The two di-cyclic aromatic hydrocarbon metabolites (∑OH-NAP), on average, contributed 83% to the ∑OH-PAH in mass. This is comparable with the 82% in the U.S. population from the NHANES results [44]. NAP, FLU, and PHE are mostly in gaseous phase in the ambient air, while PYR is on the borderline but mainly in gaseous under relatively high ambient temperature [45]. The 1-OH-PYR only composed of about 1% of the total analyzed OH-PAHs in mass in this study. The recorded sampling temperature during the field sampling time ranged from 13.7°C to 34.9°C with an average of 27.8°C. This relatively high ambient temperature made the ambient PYR more likely to be found in the gaseous phase [46].

Compared with in-cabin PM_2.5_ and UFP, the reduction of PAH exposure by WC+HECA was not significant, which is probably due to the gaseous nature of the measured PAHs. The HECA filter was less effective for gaseous PAHs than particles. Notably, PAHs were found to be bound to airborne particles of different sizes [47], and some PAH species in particle phase (more than 4 rings) are also likely to be at high levels on road and cause health risks [48]. Therefore, the results of this study only indicate that the effect of HECA filters was limited for gas phase PAHs, but might be still effective for particle-bound and higher molecular weight PAH reduction, which was not measured in this study.

### PM/PAH exposures and health effects

The significant positive correlations between the in-cabin PM exposures and the post-test urinary MDA observed in this study support the previous findings that PM exposures induce systematic oxidative stress in human. The possible pathways linking PM exposures and urinary MDA generation include: (i) activation of leukocyte NADPH oxidase and myeloperoxidase [49]; (ii) interference with normal mitochondrial functions [50]; and (iii) depletion of antioxidant capacities [51]. Some chemical compositions of PM_2.5_ and UFPs can also contribute to the oxidative stress through other pathways. In addition, the decrease of post-test MDA level under WC+HECA suggests the potential health benefit of the tested mitigation strategy.

Previous studies have reported significant association between urinary OH-PAHs and MDA [15, 42]. But such association was not observed in this study. This is probably due to the differences in the study design. In this study, we measured the urinary MDA levels immediately after the exposures, reflecting the acute health effects, whereas most other studies on PAHs and oxidative stress association were based on long term observations. For example, one of the longitudinal studies found significant association between urinary OH-PAHs and MDA based on samples collected in several months [28]. Different from the PM, the PAHs’ capacity of inducing ROS depends on their relatively longer and more complex metabolizing processes. Given that the median half-lives of the analyzed PAHs in human body range from 2.5 to 6.1 hours, the insignificant association between OH-PAH_trap_ and MDA in our study is possibly due to the fact that the post-test samples collected right after the 6-hr driving cannot reflect the MDA generation induced by the 6-hr PAH exposures. Nevertheless the difference in our results between PAHs and PM suggest different mechanisms in inducing oxidative stress.

There are several limitations of this study. First, 17 taxis and drivers were relatively a small sample size. However, the study power was increased with repeated-measures study design, enabling each subject to serve as his/her own control. Second, no environmental PAH data were collected during the tests because of the instrument restrictions. Instead, urinary metabolites and a pharmacokinetic model were used to calculate the exposure surrogates, which may induce uncertainties. Specifically, only population mean elimination rates were available and used to calculate the OH-PAH_trap_, and individual variations in PAH metabolism among the drivers were not taken into consideration. However, this interference from individual variation could be at least partially controlled by the repeated-measures design. The elimination rates in the literature from both dietary and inhalation intake were compared in Table C in S1 File. Although the calculated OH-PAH_trap_ values were different between dietary and inhalation routes, the association between the OH-PAH_trap_ and MDA remains insignificant. Finally, the concentration of MDA could also be influenced by factors other than the PM through inhalation, such as other inhalable pollutants and diet. However, the post-test MDA shows a similar trend with the in-cabin PM_2.5_ and UFP suggesting PM exposure may play an important role in affecting MDA levels.

To our knowledge, this is the first intervention study using repeated-measures design to examine the effectiveness of various mitigation strategies and the health effects of TRAPs on taxi drivers. HECA filters were found to reduce both PM_2.5_ and UFP levels significantly inside taxis. The positive association between the in-cabin PM levels and the drivers’ urinary MDA concentrations shows that oxidative stress is a possible mechanism for the adverse health effects associated with TRAP exposures.

## Supporting information

S1 File (DOCX)

S1 Fig (TIF)
