# Supplementary material for: High efficiency cabin air filter in vehicles reduces drivers' roadway particulate matter exposures and associated lipid peroxidation
Source: PLoS One. 2017 Nov 27;12(11):e0188498. doi: 10.1371/journal.pone.0188498 (PMC5703570; doi:10.1371/journal.pone.0188498)
Supplement: S1 File — (DOCX) [file pone.0188498.s001.docx]

**Supporting Information**

**High Efficiency Cabin Air Filter in Vehicles Reduces Drivers' Roadway Particulate Matter Exposures and Associated Lipid Peroxidation**

Nu Yu^1^, Shi Shu^1^, Yan Lin^1^_,_ Jianwen She^2^, Ho Sai Simon Ip^2^, Xinghua Qiu^3^, and Yifang Zhu^1*^

1. Department of Environmental Health Sciences, Jonathan and Karin Fielding School of Public Health, University of California, Los Angeles, California 90095, United States
2. California Department of Public Health, 850 Marina Bay Parkway, Richmond, California 94804, United States
3. State Key Joint Laboratory for Environmental Simulation and Pollution Control, College of Environmental Sciences and Engineering and Center for Environment and Health, Peking University, Beijing 100871, People’s Republic of China

* Corresponding Author: Yifang Zhu, Tel: 310-825-4324, Email: [yifang@ucla.edu](mailto:yifang@ucla.edu)

**Table A** Summary of OH-PAH changes under different test conditions

| **Mitigation** | **NM** | **WC** | **WC+HECA** |
| --- | --- | --- | --- |
| ***Increment*** |  |  |  |
| 1-OH-NAP | 0.47±0.58 | 0.25±0.25  (-47%) | 0.31±0.29  (-34%) |
| 2-OH-NAP | 1.55±1.26 | 1.23±1.09  (-21%) | 1.11±0.80  (-28%) |
| ∑OH-NAP | 2.02±1.48 | 1.48±1.29  (-27%) | 1.42±1.10  (-30%) |
| 2-OH-FLU | 0.70±0.69 | 0.45±0.21  (-36%) | 0.50±0.25  (-29%) |
| 3-OH-FLU | 0.15±0.16 | 0.11±0.07  (-27%) | 0.10±0.07  (-33%) |
| 9-OH-FLU | 0.96±0.70 | 0.75±0.58  (-22%) | 0.79±0.41  (-18%) |
| ∑OH-FLU | 1.81±1.47 | 1.33±0.69  (-27%) | 1.39±0.76  (-23%) |
| 1-OH-PHE | 0.23±0.28 | 0.15±0.07  (-35%) | 0.21±0.12  (-9%) |
| 2-OH-PHE | 0.11±0.09 | 0.08±0.05  (-27%) | 0.10±0.06  (-9%) |
| 3-OH-PHE | 0.17±0.20 | 0.12±0.07  (-29%) | 0.13±0.08  (-24%) |
| ∑OH-PHE | 0.51±0.44 | 0.35±0.16  (-31%) | 0.44±0.22  (-14%) |
| 1-OH-PYR | 0.28±0.43 | 0.18±0.13  (-36%) | 0.21±0.21  (-25%) |
| ***Pre-test*** |  |  |  |
| 1-OH-NAP | 3.48±5.22 | 2.30±2.71  (-34%) | 2.24±3.04  (-36%) |
| 2-OH-NAP | 5.86±4.86 | 5.71±4.65  (-2%) | 5.13±4.33  (-12%) |
| ∑OH-NAP | 9.34±8.85 | 8.02±4.99  (-14%) | 7.37±6.20  (-21%) |
| 2-OH-FLU | 0.36±0.48 | 0.22±0.10  (-44%) | 2.71±3.89  (-26%) |
| 3-OH-FLU | 0.16±0.15 | 0.11±0.07  (-31%) | 0.18±0.33  (8%) |
| 9-OH-FLU | 0.47±0.34 | 0.42±0.22  (-11%) | 0.48±0.41  (1%) |
| ∑OH-FLU | 1.00±0.87 | 0.73±0.29  (-26%) | 0.92±1.08  (-8%) |
| 1-OH-PHE | 0.19±0.18 | 0.16±0.14  (-15%) | 0.16±0.12  (-15%) |
| 2-OH-PHE | 0.08±0.08 | 0.05±0.03  (-29%) | 0.07±0.09  (-13%) |
| 3-OH-PHE | 0.13±0.18 | 0.08±0.05  (-37%) | 0.10±0.15  (-20%) |
| ∑OH-PHE | 0.39±0.43 | 0.29±0.20  (-25%) | 0.33±0.32  (-17%) |
| 1-OH-PYR | 0.14±0.12 | 0.13±0.09  (-5%) | 0.12±0.10  (-13%) |
| ***Post-test*** |  |  |  |
| 1-OH-NAP | 2.94±3.85 | 1.78±1.91  (-40%) | 1.97±2.19  (-33%) |
| 2-OH-NAP | 5.80±4.53 | 5.05±4.63  (-13%) | 4.46±3.03  (-23%) |
| ∑OH-NAP | 8.74±6.74 | 6.82±4.61  (-22%) | 6.44±3.82  (-26%) |
| 2-OH-FLU | 0.31±0.32 | 0.20±0.08  (-36%) | 0.23±0.16  (-26%) |
| 3-OH-FLU | 0.15±0.14 | 0.10±0.06  (-33%) | 0.13±0.14  (-13%) |
| 9-OH-FLU | 0.43±0.28 | 0.37±0.24  (-14%) | 0.39±0.22  (-9%) |
| ∑OH-FLU | 0.89±0.64 | 0.67±0.32  (-25%) | 0.75±0.46  (-15%) |
| 1-OH-PHE | 0.17±0.17 | 0.13±0.08  (-24%) | 0.15±0.07  (-12%) |
| 2-OH-PHE | 0.06±0.05 | 0.05±0.02  (-17%) | 0.06±0.04  (0%) |
| 3-OH-PHE | 0.11±0.14 | 0.08±0.04  (-27%) | 0.09±0.06  (-18%) |
| ∑OH-PHE | 0.35±0.36 | 0.26±0.12  (-24%) | 0.30±0.16  (-14%) |
| 1-OH-PYR | 0.15±0.19 | 0.12±0.08  (-20%) | 0.12±0.09  (-20%) |

Abbreviations: NM=No mitigation, WC=Window closed, HECA=High Efficiency Cabin Air Filter in Use.

Figures in parenthesis under WC and WC+HECA indicate percentage of change from non-mitigation. Data expressed as mean ± SD (N=17).

No significance detected from paired t-test comparing with (NM).

**Table B** Pearson’s correlation coefficients (r) among PM_2.5_, UFP, pre- and post-test OH-PAHs, and OH-PAH_trap_

|  | **PM_2.5_** | | **UFP** | **Pr^a^∑OH-NAP** | **Pr∑OH-FLU** | **Pr∑OH-PHE** | **Pr1-OHPYR** | **Po^b^∑OH-NAP** | **Po∑OH-FLU** | **Po∑OH-PHE** | **Po1-OH-PYR** | **∑OH-NAP_trap_^c^** | **∑OH-FLU_trap_** | **∑OH-PHE_trap_** | **1-OH-PYR_trap_** |
| --- | --- | --- | --- | --- | --- | --- | --- | --- | --- | --- | --- | --- | --- | --- | --- |
| **PM_2.5_** | **1** | | 0.22 | 0.18 | 0.02 | 0.00 | 0.09 | 0.15 | 0.04 | 0.01 | 0.04 | 0.09 | 0.06 | 0.04 | 0.02 |
| **UFP** |  | | **1** | 0.01 | 0.05 | 0.26*^d^ | 0.24* | 0.04 | 0.02 | 0.24* | 0.26* | 0.03 | 0.08 | 0.16 | 0.23* |
| **Pr∑OH-NAP** |  |  | | **1** | **0.59^e^*** | 0.36* | 0.42* | **0.88*** | **0.57*** | 0.26* | 0.21 | **0.64*** | 0.41* | 0.11 | 0.08 |
| **Pr∑OH-FLU** |  |  | |  | **1** | **0.73*** | **0.6*** | 0.45* | **0.79*** | **0.63*** | 0.45* | 0.38* | **0.60*** | 0.40* | 0.31* |
| **Pr∑OH-PHE** |  |  | |  |  | **1** | **0.81*** | 0.31* | 0.49* | **0.87*** | **0.69*** | 0.26* | 0.20 | **0.57*** | **0.57*** |
| **Pr1-OH-PYR** |  |  | |  |  |  | **1** | 0.46* | **0.50*** | **0.73*** | **0.8*** | 0.44* | 0.24* | 0.46* | **0.60*** |
| **Po∑OH-NAP** |  |  | |  |  |  |  | **1** | **0.6*** | 0.32* | 0.33* | **0.92*** | **0.53*** | 0.23* | 0.24* |
| **Po∑OH-FLU** |  |  | |  |  |  |  |  | **1** | **0.57*** | 0.48* | **0.61*** | **0.93*** | 0.49* | 0.37* |
| **Po∑OH-PHE** |  |  | |  |  |  |  |  |  | **1** | **0.87*** | 0.34* | 0.38* | **0.87*** | **0.81*** |
| **Po1-OH-PYR** |  |  | |  |  |  |  |  |  |  | **1** | 0.41* | 0.33* | **0.82*** | **0.93*** |
| **∑OH-NAP_trap_** |  |  | |  |  |  |  |  |  |  |  | **1** | **0.62*** | 0.32* | 0.35* |
| **∑OH-FLU_trap_** |  |  | |  |  |  |  |  |  |  |  |  | **1** | **0.50*** | 0.38* |
| **∑OH-PHE_trap_** |  |  | |  |  |  |  |  |  |  |  |  |  | **1** | **0.90*** |
| **1-OH-PYR_trap_** |  |  | |  |  |  |  |  |  |  |  |  |  |  | **1** |

^a^Pr=pre-test, ^b^Po=post-test, ^c^OH-PAH_trap_=calculated urinary OH-PAH increment due to TRAP exposure

^d^* shows the significance of associations (p<0.05), ^e^r values are highlighted in bold when > 0.5.

**Table C** Comparison of calculated OH-PAH_trap_ with dietary and inhalation intake half-lives

|  | | **Literature 1**  **(Ref. of this study)** | **Literature 2** | **Literature 3 (Smoker study)** |
| --- | --- | --- | --- | --- |
|  | | Li et. al. [37] | Motorykin et. al. [52] | St Helen et. al. [53] |
| **Intake route** | | ***dietary*** | ***dietary*** | ***inhalation*** |
| ***Reported half-life (hr) / elimination rate (k)*** | 1-NAP | 4.3 / 0.16 | 3.4 / 0.21 | *8.6 / 0.08** |
|  | 2-NAP | 2.5 / 0.27 | 2.4 / 0.28 | 9.4 / 0.07 |
|  | 2-FLU | 2.9 / 0.24 | 2.6 / 0.26 | 4.1 / 0.17 |
|  | 3-FLU | 6.1 / 0.11 | 7.0 / 0.10 | 8.2 / 0.08 |
|  | 9-FLU | 3.1 / 0.23 | 1.7 / 0.41 | *6.2 / 0.11** |
|  | 1-PHE | 5.1 / 0.14 | 3.1 / 0.22 | *10.2 / 0.07** |
|  | 2-PHE | 3.9 / 0.18 | 3.7 / 0.19 | *7.8 / 0.09** |
|  | 3-PHE | 4.1 / 0.17 | 2.6 / 0.27 | *8.2 / 0.08** |
|  | 1-PYR | 3.9 / 0.18 | 4.4 / 0.16 | 6.0 / 0.12 |
| ***Calculated OH-PAHtrap*** | | | | |
| **∑OH-NAP_trap_**  **(µg/g cr)** | NM | 4.69 | 5.27 | 2.24 |
|  | WC | 3.47 | 4.06 | 0.63 |
|  | WC+HECA | 4.40 | 3.94 | 1.39 |
| **∑OH-FLU_trap_**  **(µg/g cr)** | NM | 0.51 | 0.57 | 0.45 |
|  | WC | 0.40 | 0.46 | 0.35 |
|  | WC+HECA | 0.48 | 0.50 | 0.43 |
| **∑OH-PHE_trap_**  **(µg/g cr)** | NM | 0.15 | 0.20 | 0.10 |
|  | WC | 0.12 | 0.17 | 0.08 |
|  | WC+HECA | 0.18 | 0.19 | 0.10 |
| **1-OH-PYR_trap_**  **(µg/g cr)** | NM | 0.05 | 0.05 | 0.02 |
|  | WC | 0.05 | 0.04 | 0.01 |
|  | WC+HECA | 0.07 | 0.03 | 0.03 |

*estimated values from Literature 1 by doubling the half-lives.

**Section A. Pharmacokinetic model for estimating the 6-hr PAH exposure**

The concentration of post-test OH-PAHs is expected to depend on both PAH exposure during the 6-hr monitored test and the pre-test level of OH-PAHs (i.e. pre-test OH-PAHs concentration). Since both pre-and post-test OH-PAHs levels were measured in this study, a pharmacokinetic model can be used to calculate PAH exposure during the 6-hour monitored test.

Previous studies indicated that elimination rate constants for urinary OH-PAH were first-order.[37] Assuming the urinary OH-PAH increment rate due to the continuous 6-hr TRAP exposure is *E_i_*, the alteration of OH-PAHs in urine could be described as:

${dc}_{i}/dt=E_{i}-kC_{i}$ (S1)

Where *i* is an index for each driver, *C_i_* is the concentration of OH-PAHs in urine, and *k* is the urine elimination rate of OH-PAHs in human body. Integrate equation (S1) and we get:

$C_{i}\left( t \right)=C_{i}\left( 0 \right)e^{-kt}+E_{i}(1-e^{-kt})/k$ (S2)

where *C_i_* (0) and *C_i_* (*t*) are the OH-PAH concentrations for driver *i* at initial condition (*t*=0) and time *t*, respectively. S1 Fig illustrates the two terms of equation (S2) and the combination, where *C_i_* (0) *e ^-kt^* indicates the first order decay of the OH-PAH concentration from the initial condition, and *E_i_* (1 - *e ^-kt^* ) / *k* indicates the OH-PAH concentration increment due to the continuous TRAP exposure during the 6-hr monitored test.

We set the pre-test concentration as the initial condition, and calculated the urinary OH-PAH increment at *t*=6 hour due to the TRAP exposure (*C_i,trap_*) from equation:

$C_{i,trap}=E_{i}\left( 1-e^{-kt} \right)/k=C_{i,post}-C_{i,pre}e^{-6k}$ (S3)

In this equation, *C_i,pre_* and *C_i,post_* were measured, the value of $k$ was obtained from previous studies for each OH-PAH species [37]. Thus, the increment of each and total OH-PAH (OH-PAH_trap_ and ∑OH-PAH_trap_) due to the 6-hr monitored TRAP exposure can be calculated.

**References**

37. Li Z, Romanoff L, Bartell S, Pittman EN, Trinidad DA, McClean M, et al. Excretion Profiles and Half-Lives of Ten Urinary Polycyclic Aromatic Hydrocarbon Metabolites after Dietary Exposure. Chemical Research in Toxicology. 2012;25(7):1452-61.

52. Motorykin O, Santiago-Delgado L, Rohlman D, Schrlau JE, Harper B, Harris S, et al. Metabolism and excretion rates of parent and hydroxy-PAHs in urine collected after consumption of traditionally smoked salmon for Native American volunteers. Science of the Total Environment. 2015;514:170-7.

53. St Helen G, Goniewicz ML, Dempsey D, Wilson M, Jacob P, Benowitz NL. Exposure and Kinetics of Polycyclic Aromatic Hydrocarbons (PAHs) in Cigarette Smokers. Chemical Research in Toxicology. 2012;25(4):952-64.
